# Mapping Modeled Exposure of Wildland Fire Smoke for Human Health Studies in California

**DOI:** 10.3390/atmos10060308

**Published:** 2019-06-04

**Authors:** Patricia D. Koman, Michael Billmire, Kirk R. Baker, Ricardo de Majo, Frank J. Anderson, Sumi Hoshiko, Brian J. Thelen, Nancy H.F. French

**Affiliations:** 1 Environmental Health Sciences, School of Public Health, University of Michigan, Ann Arbor, MI 48109, USA; 2 Michigan Tech Research Institute, Michigan Technological University, Ann Arbor, MI, 48105 USA;; 3 Office of Air Quality Planning & Standards, Office of Air and Radiation, U.S. Environmental Protection Agency, Research Triangle Park, NC, 27709 USA;; 4 Health Behavior Health Education, University of Michigan School of Public Health, Ann Arbor, MI 48109, USA;; 5 Obstetrics and Gynecology, University of Michigan School of Medicine, Ann Arbor, MI 48109, USA;; 6 Environmental Health Investigations Branch, California Department of Public Health, Richmond, CA 94804,USA;

**Keywords:** wildland fire, air quality, exposure, particulate matter, geospatial analysis, public health, chemical transport model, atmospheric modeling, epidemiology

## Abstract

Wildland fire smoke exposure affects a broad proportion of the U.S. population and is increasing due to climate change, settlement patterns and fire seclusion. Significant public health questions surrounding its effects remain, including the impact on cardiovascular disease and maternal health. Using atmospheric chemical transport modeling, we examined general air quality with and without wildland fire smoke PM_2.5_. The 24-h average concentration of PM_2.5_ from all sources in 12-km gridded output from all sources in California (2007–2013) was 4.91 μg/m^3^. The average concentration of fire-PM_2.5_ in California by year was 1.22 μg/m^3^ (~25% of total PM_2.5_). The fire-PM_2.5_ daily mean was estimated at 4.40 μg/m^3^ in a high fire year (2008). Based on the model-derived fire-PM_2.5_ data, 97.4% of California’s population lived in a county that experienced at least one episode of high smoke exposure (“smokewave”) from 2007–2013. Photochemical model predictions of wildfire impacts on daily average PM_2.5_ carbon (organic and elemental) compared to rural monitors in California compared well for most years but tended to over-estimate wildfire impacts for 2008 (2.0 μg/m^3^ bias) and 2013 (1.6 μg/m^3^ bias) while underestimating for 2009 (−2.1 μg/m^3^ bias). The modeling system isolated wildfire and PM_2.5_ from other sources at monitored and unmonitored locations, which is important for understanding population exposure in health studies. Further work is needed to refine model predictions of wildland fire impacts on air quality in order to increase confidence in the model for future assessments. Atmospheric modeling can be a useful tool to assess broad geographic scale exposure for epidemiologic studies and to examine scenario-based health impacts.

## 1. Introduction

An understudied and growing source of air pollution is smoke from wildland fires. Wildland fires—including unplanned wildfires, prescribed fires, and agricultural burning—have increased rapidly in the U.S. over the past three decades, accounting for 40% of PM emissions inventories in the U.S. [1,2]. Despite the growing recognition of the impact of wildfire on health and its association with general ambient particulate matter (PM) and respiratory and cardiovascular disease, scientific evidence is lacking for wildfire smoke associations with outcomes other than asthma and chronic obstructive pulmonary disease [3–5]. More specifically, studies of single fires in California have reported limited associations between wildfire PM exposure and cardiovascular hospitalizations [6,7] and reductions in birthweight [8]. Wildland fire smoke exposure affects a broad proportion of the U.S. population and the number of those affected is increasing [2,9]. The growing wildland-urban interface includes nearly 39% of all housing units, with an estimated 46 million persons in the Western U.S. alone, including an estimated half a million pregnant women, exposed to wildfire smoke [10–12]. Ecologists recognize fire’s permanence in biologic cycles and the likely increases of its occurrence with climate change, population settlement patterns, and fire seclusion. Accordingly, novel studies examining multi-fire periods with additional wildfire exposure metrics are needed to characterize associations in vulnerable groups and to establish a scientific basis for action to minimize smoke exposure. One reason for the limited number of epidemiologic studies of wildland fire air emissions is related to the need for better exposure assessment techniques, including modeling, stationary monitoring, remote sensing, or low cost sensors [3,13,14]. The purposes of this study are to estimate county level exposures to wildland fire-PM for California and to examine the strengths and limitations of using chemical transport models for health studies.

Although air quality affected by wildland fire smoke could be classified as an “exceptional event” and excluded from calculating exceedances of national ambient air quality standards, it is important to study the effects of human exposure to smoke. The link between wildland fire smoke and adverse health effects is supported by a larger body of general ambient air pollution studies [15,16]. PM and ozone are associated with a suite of cardiopulmonary health outcomes, with PM in particular associated with cardiovascular [17] and maternal/birth endpoints [18–22]. Air pollution is hypothesized to induce systemic oxidative stress and inflammation, which are pathways in the pathogenesis of respiratory and cardiovascular disease [17,23].

Because fine particulate air pollution (PM_2.5_) is a multi-dimensional pollutant originating from a variety of sources, including wildland fire, there are gaps in our understanding of how exposure to wildland fire smoke impacts health. While wildfires produce high levels of air pollutants, the chemical signature differs from other source of ambient PM and shows differential toxicity for some endpoints [24–27]. Exposure scenarios differ as well, with substantially sharper peaks during wildfires [7]. PM_2.5_ concentrations during several California wildfires has been estimated between 3 to 31 times the daily PM_2.5_ standard concentration established by the U.S. Environmental Protection Agency (EPA) [28].

While fire-specific PM exposure is associated with pulmonary outcomes, the evidence for cardiovascular disease is mixed, and there is evidence with limitations regarding maternal and birth outcomes [3,4,8,29]. Increases in exposure to air pollutants during pregnancy have been positively associated with adverse birth outcomes and an increased risk of pregnancy-induced hypertensive disorders [22,30]. Pregnancy-induced hypertensive disorders can lead to maternal and perinatal morbidity and mortality, but the causes are not well understood [31,32] and the contribution of wildland fire smoke has not been widely studied. Associations between general ambient PM_2.5_ and hypertensive disorders of pregnancy (HDP) are more consistent with upper percentile concentrations and pollutants whose distributions contain geo-temporal spikes, much like wildland fire distributions, rather than the more chronic low-level exposures typifying ambient air pollution [33,34].

Some populations are disproportionately affected by wildfire smoke based on their susceptibility, geographic location, or smoke exposure characteristics. A national study showed via principal component analyses that several vulnerability factors similar to those for general air pollution were relevant for fire-PM_2.5_; specifically, factors included age (e.g., those >65 years); adults with respiratory disease (e.g., COPD and asthma); adults with hypertension, obesity and diabetes; children with asthma; and economic deprivation [35]. Age may also be a factor contributing to a population’s susceptibility [36–40].

In addition to underlying health status of a population, health impacts of wildfire smoke may be related to characteristics of the smoke exposure. These characteristics include the chemical composition, pollutant concentrations, the intensity of the fire, timing, and duration of smoke exposure; land features that contribute to smoke dispersal or contact with populations; extent to which populations can protect themselves from the exposure; and the number of individuals who are exposed. Furthermore, vulnerability factors may influence exposure; these include place-based characteristics (e.g., proximity to the wildland-urban interface, housing density and ventilation, low SES [35]), and fire-based characteristics (e.g., combustion characteristics, vegetation type, prescribed vs. wildfire [41]). However, there remain serious knowledge gaps about the full effects of these vulnerability factors and wildfire smoke exposures.

## 2. Materials and Methods

We used the community multiscale air quality(CMAQ;https://www.epa.gov/cmaq), photochemical transport model in order to conduct multiple annual air quality simulations [42,43]. CMAQ is a three-dimensional grid-based model that simulates chemical and physical processes in each grid cell and uses Eulerian diffusion and transport processes to move chemical species to other grid cells [44]. CMAQ combines emissions from both natural (e.g., wildfire smoke) and anthropogenic (e.g., point-source industrial, automotive) sources with weather-based atmospheric transport, dispersion, chemical transformation, and deposition using time and space variant meteorology [45]. By conducting CMAQ model runs with and without wildfire smoke emissions sources, we can characterize the relative impact of wildfire smoke on ambient air quality.

**Figure 1.**
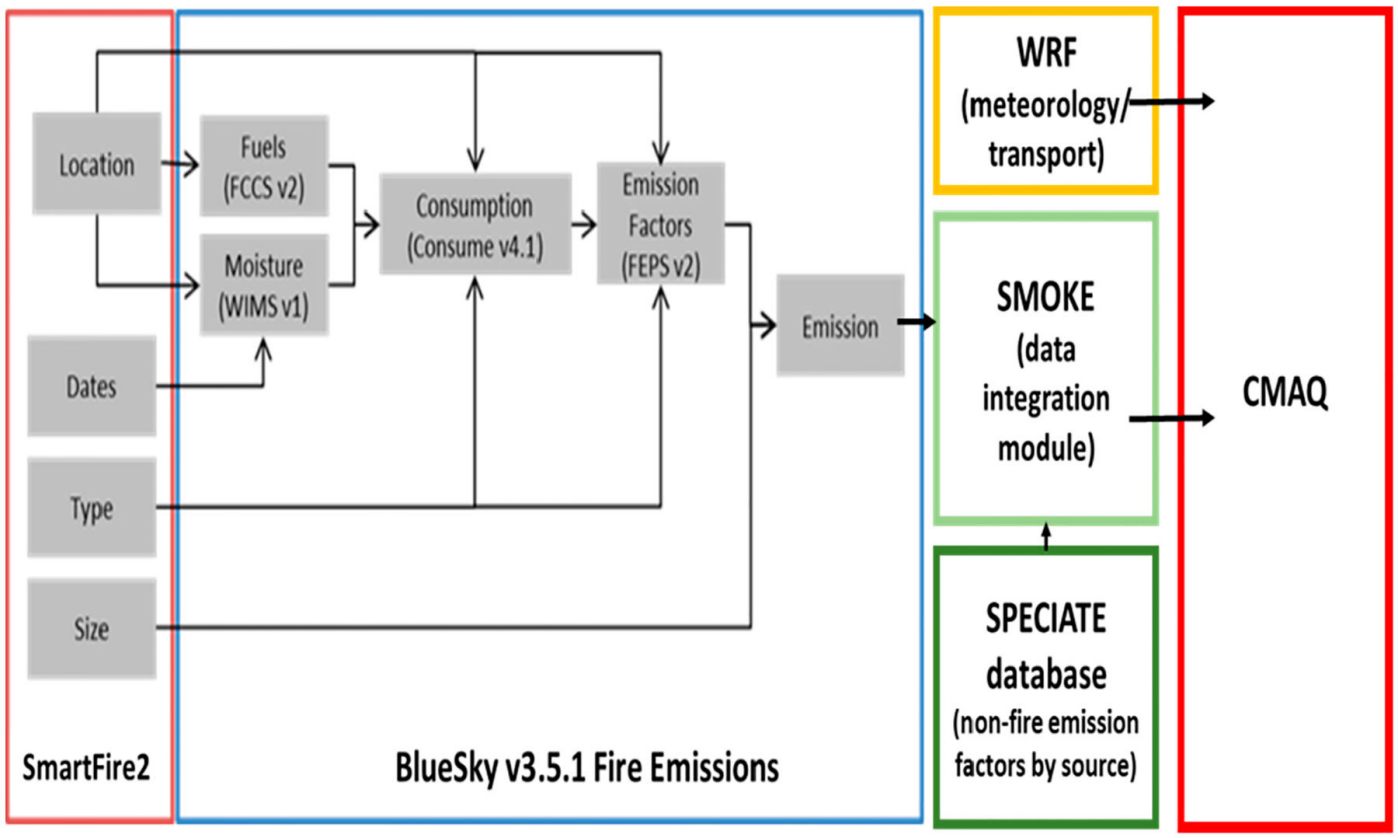
The fire-related emissions components (SmartFire2 and BlueSky v 3.5.1, orange and blue boxes) are combined with meteorology (yellow box), emissions from other sources (SMOKE) and their chemical composition (SPECIATE) (green boxes) as inputs to the Community Multiscale Air Quality (CMAQ) chemical transport models (red box). Key modeling elements include the following: SmartFire2; FCCS: Fuel Characteristic Classification System [49]; WIMS: Weather Information Management System [52]; Consume [53]; FEPS: Fire Emission Production Simulator [54]; WRF: Weather Research and Forecasting model [55]; SMOKE: Sparse Matrix Operator Kernel Emissions modeling system [56]; SPECIATE [57]; CMAQ: Community Multiscale Air Quality [42,43].

For this study, all non-fire-source emissions (based on National Emission Inventories) and fire event information were modeled with the CMAQ model v5.0.1/5.0.2 [46]. Wildfire emission sources for input to CMAQ were modeled using the BlueSky (v3.5.1) framework [47] (Figure 1). The approach requires quantification of four parameters: area burned (a.k.a., fire activity), fuel loading (biomass per unit area), the fraction of biomass fuel consumed by fire, referred to also as fuel consumption or combustion completeness, and emission factors [47]. For fire date, size, type, and location data, BlueSky uses the SmartFire2 fire information system, which aggregates and reconciles a comprehensive set of disparate wildfire information sources. SmartFire2 sources include satellite detections, daily situation reports (ICS-209 reports produced by incident managers), and GEOMAC perimeters for U.S. wildland and prescribed burns as well as burns (>100 acres) [48]. Fuel Characteristic Classification System (FCCS) provides spatially-defined fuel type and loading data [49]. Fuel consumption and resulting emissions are calculated using Consume v4.1 [50] with fuel type, loading and fuel moisture (via weather information management system—WIMS; https://famit.nwcg.gov/applications/WIMS) as inputs. We did not adjust CMAQ modeling outputs using air pollution monitoring data, satellite data or other techniques; instead we compared the modeling output to speciated monitored data [51].

This study analyzed CMAQ-modeled daily PM_2.5_ concentration estimates for the years 2007–2013 for the state of California at 12-km spatial resolution from a national run (24-h period is from midnight to midnight, adjusted for time zone). California experienced particularly active fire seasons (>750,000 acres burned) in 2007, 2008, and 2012. To obtain fire-specific estimates, separate CMAQ model runs were performed with and without wildfire emissions sources, and fire-PM_2.5_ was calculated by subtracting the former from the latter. We examined CMAQ output at the more finely resolved grid-cell level and aggregated to the county level.

**Figure 2.**
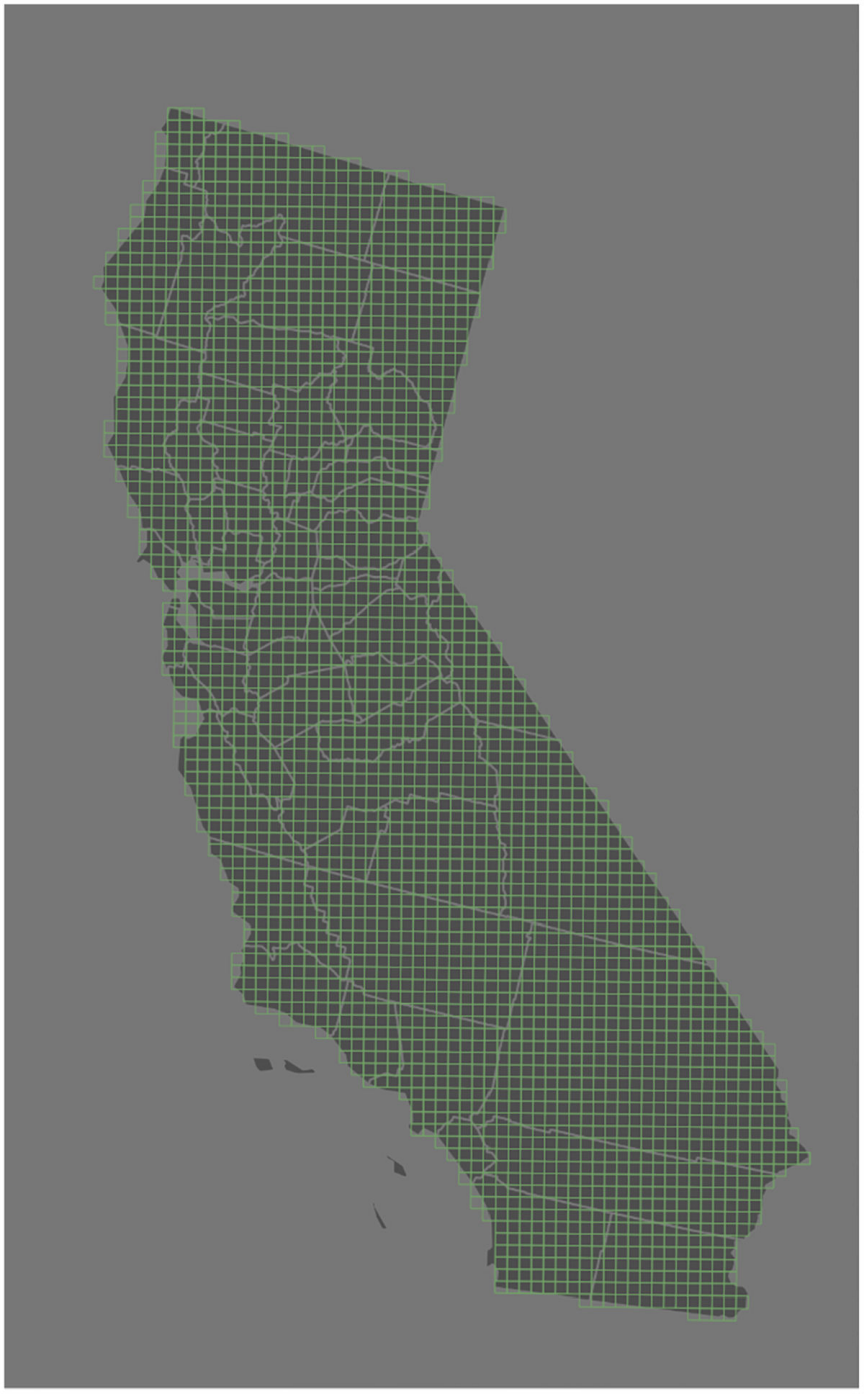
CMAQ 12 km grid (light green lines) overlaid onto California county boundaries (gray lines). County-level statistics were derived by summarizing CMAQ-derived values for each grid cell whose centroid fell within the county boundary.

Daily county-level mean fire-PM_2.5_ concentrations were calculated by averaging values of all CMAQ grid cells whose centroid fell within the county boundary (Figure 2) for that day. From these daily county averages, we calculated county-level mean annual fire-PM_2.5_ concentration for each year (2007–2013). From these annual county averages, we calculated quartile breaks that were used to bin county populations into fire-PM_2.5_ exposure classes, similar to a national study by Rappold and colleagues [35]. Because there is no conclusive evidence of a threshold for response to PM_2.5_ and little evidence of a demarcation of healthy v. “unhealthy” fire-related PM_2.5_, we used quartiles of annual air pollution to compare population exposure at different levels. We compared statewide to national analyses [35].

To assess model performance, daily average PM_2.5_ measurements of elemental and organic carbon from the interagency monitoring of protected visual environments (IMPROVE) monitor network were used to evaluate modeling system tendencies toward over- or underprediction of wildfire impacts in the different years simulated. IMPROVE monitors were chosen because this network provides speciation and is largely rural, and thus would be less impacted by biases related to the characterization of urban areas. Previous studies of CMAQ model performance have assessed PM_2.5_ prediction in populated urban area [43,58,59].

We matched ambient monitored measurements with the model grid cell where the monitor was located. Daily average comparisons were aggregated for each model simulation year. We calculated these comparisons for the top three quartiles (fire-impacted areas) and for the lowest quartile of fire-PM_2.5_ concentration (little or no fire areas) using the quartile breaks as described above.

Because wildfires are typically short-lived events (e.g., several days) that often elevate PM_2.5_ to several orders of magnitude above ambient levels, we performed two types of analyses to the modeled air pollution. First, we plotted the mean fire-PM_2.5_ concentration by day for the largest 10 counties by geographic area. Second, we developed an exposure metric to reflect peak exposure patterns based on the concept of a “smokewave”, analogous to a heatwave [60]. This metric is defined here as a period when daily fire-PM_2.5_ concentration exceeds the NAAQS 24-h PM_2.5_ level of 35 μg/m^3^ for more than two consecutive days. The number of smokewave periods was calculated for each CMAQ grid cell and county so that the total population exposed to smokewaves could be estimated.

We obtained demographic and health variables from the U.S. Census for these factors at the census tract level for California for the period 2007–2013. We obtained asthma emergency department visits and hospitalizations for heart attack from the Centers for Disease Control (CDC) Environmental Public Health Tracking Network (https://ephtracking.cdc.gov/DataExplorer/#/ accessed March 3, 2018) and live births from CDC Wonder database (Source: https://wonder.cdc.gov/natality.html, accessed March 3, 2018). We compared the geospatial location of populations with these factors by quartiles of fire-PM_2.5_ for 2007–2013.

## 3. Results

### 3.1. Mean Annual Fire-PM_2.5_ Concentrations

**Figure 3.**
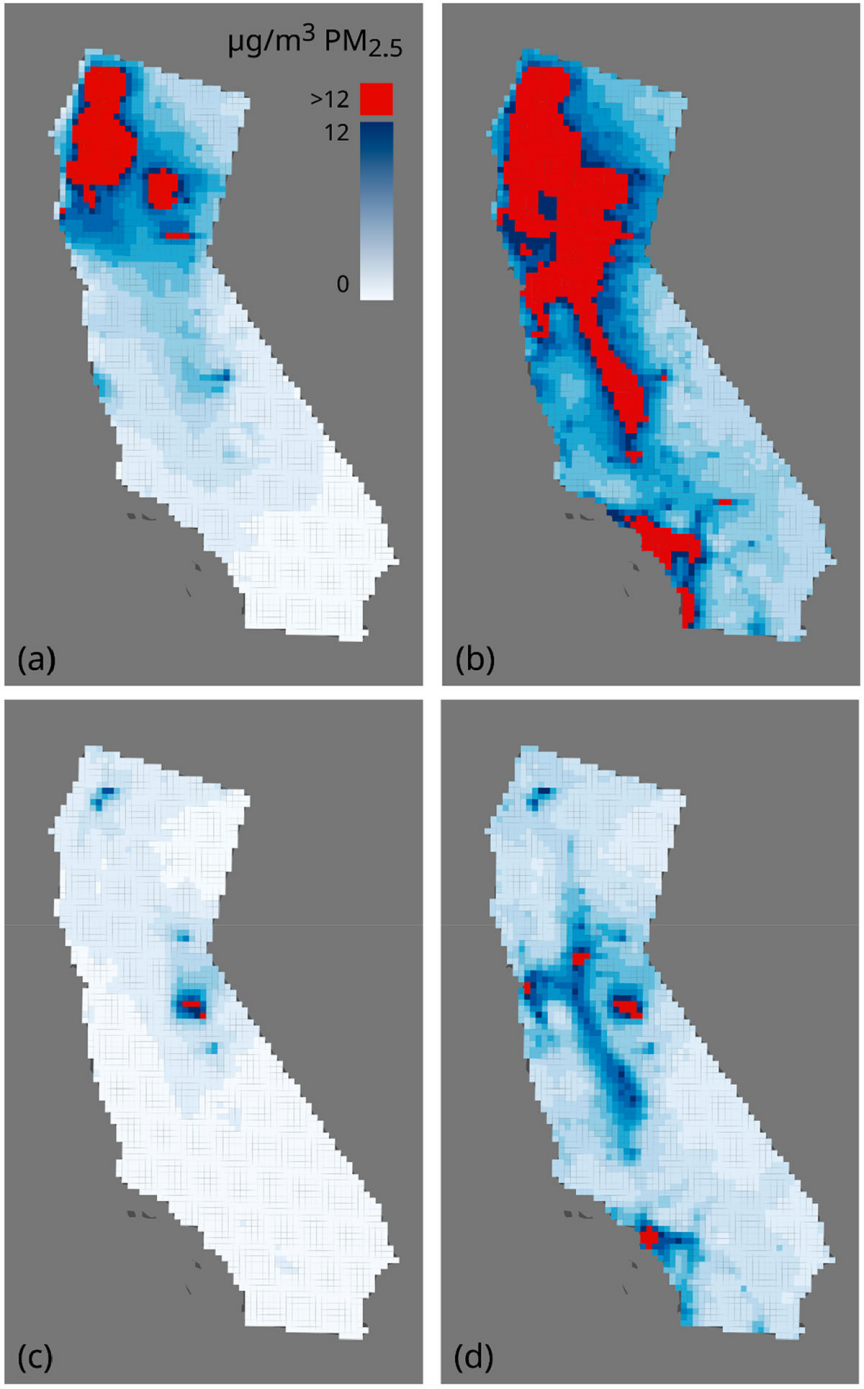
CMAQ-modeled mean annual PM_2.5_ for California by 12-km grid: (**a**) Year 2008, fire-only emissions sources; (**b**) Year 2008, all sources; (**c**) Year 2013, fire-only sources; and (**d**) Year 2013, all sources. 12 μg/m^3^ represents the National Ambient Air Quality Standards (NAAQS) level for mean annual all source PM_2.5_.

We summarized the CMAQ modeling output county level fire-PM_2.5_ by year (Table 1). Generally, PM_2.5_ emissions are declining during this period due to Clean Air Act regulations of stationary and mobile sources, but California has non-attainment areas that do not meet current health-based ambient air quality standards. The maps (Figure 3a–d) show geographical extent of fire-PM_2.5_ annual mean concentrations for selected years illustrating a high fire year (2008) and a lower fire year and low all-source PM_2.5_ (2013).

The 24-h average concentration of ambient modeled PM_2.5_ from all sources in California (2007–2013) was 4.91 μg/m^3^ (standard deviation 4.04 μg/m^3^). The yearly all-source-PM_2.5_ daily mean ranged from 3.74 μg/m^3^ (2013) to 8.90 μg/m^3^ (2008). The 24-h average concentration of fire-PM_2.5_ in California by year was 1.22 μg/m^3^ (standard deviation 3.78 μg/m^3^) accounting for about a quarter of all-source-PM_2.5_ concentrations. The annual average of the fire-PM_2.5_ daily mean ranged from 0.31 μg/m^3^ (2010) to 4.40 μg/m^3^ (2008).

The contribution of fire-PM_2.5_ to ambient PM_2.5_ in county-level averages (2007–2013) range from 4% fire-PM_2.5_ (e.g., Orange county) to 70% (e.g., Trinity County) (Table 2).

### 3.2. Populations At Risk by Annual Mean Fire-PM_2.5_ Concentration Quartiles

We estimated populations living in counties with each annual fire-PM_2.5_ exposure quartile (2007–2013) (Table 3). Based on the modeling, 23.5 million (63.4%) California residents lived in counties with >0.34 μg/m^3^ fire-PM_2.5_. Approximately 7.71 million residents (56.4%) were experiencing poverty (under twice the poverty level). Just over six million living in the top three quartiles of fire-PM_2.5_ were <18 years old (57.2%), and 2.6 million were aged 65 and older (56.5%).

More than half of the births (2007–2013) in California occurred in counties with >0.34 μg/m^3^ or in the top 3 quartiles fire-PM_2.5_. Likewise, in this same period, 75% of the asthma emergency department visits and 76% of hospitalizations for heart attacks in California occurred in counties with >0.34 μg/m^3^ fire-PM_2.5_.

### 3.3. Fire-PM_2.5_ Smokewaves: Geospatial Extent and Populations At-Risk

**Figure 4.**
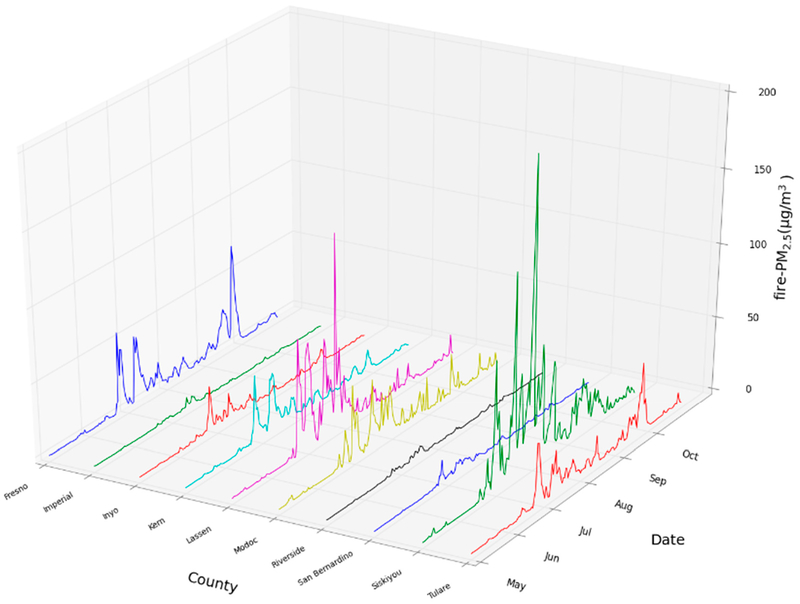
Mean daily CMAQ-derived fire-PM_2.5_ for the 10 largest counties (by area) in California from May to November 2008, illustrating spatial and temporal fluctuation in fire-PM_2.5_ concentrations.

Figure 4 shows the mean CMAQ-derived fire-PM_2.5_ concentrations by date from May to November 2008 for the ten largest California counties by land area. The pattern illustrates that for many counties, there are near zero fire-PM_2.5_ levels for many days followed by peaks during a fire incident that an annual average might mask. Accordingly, we analyzed populations residing in counties experiencing smokewave days. Furthermore, the timing and intensity of peak fire-PM_2.5_ varies across the state during this period, which relates to the challenges of siting stationary monitors.

**Figure 5.**
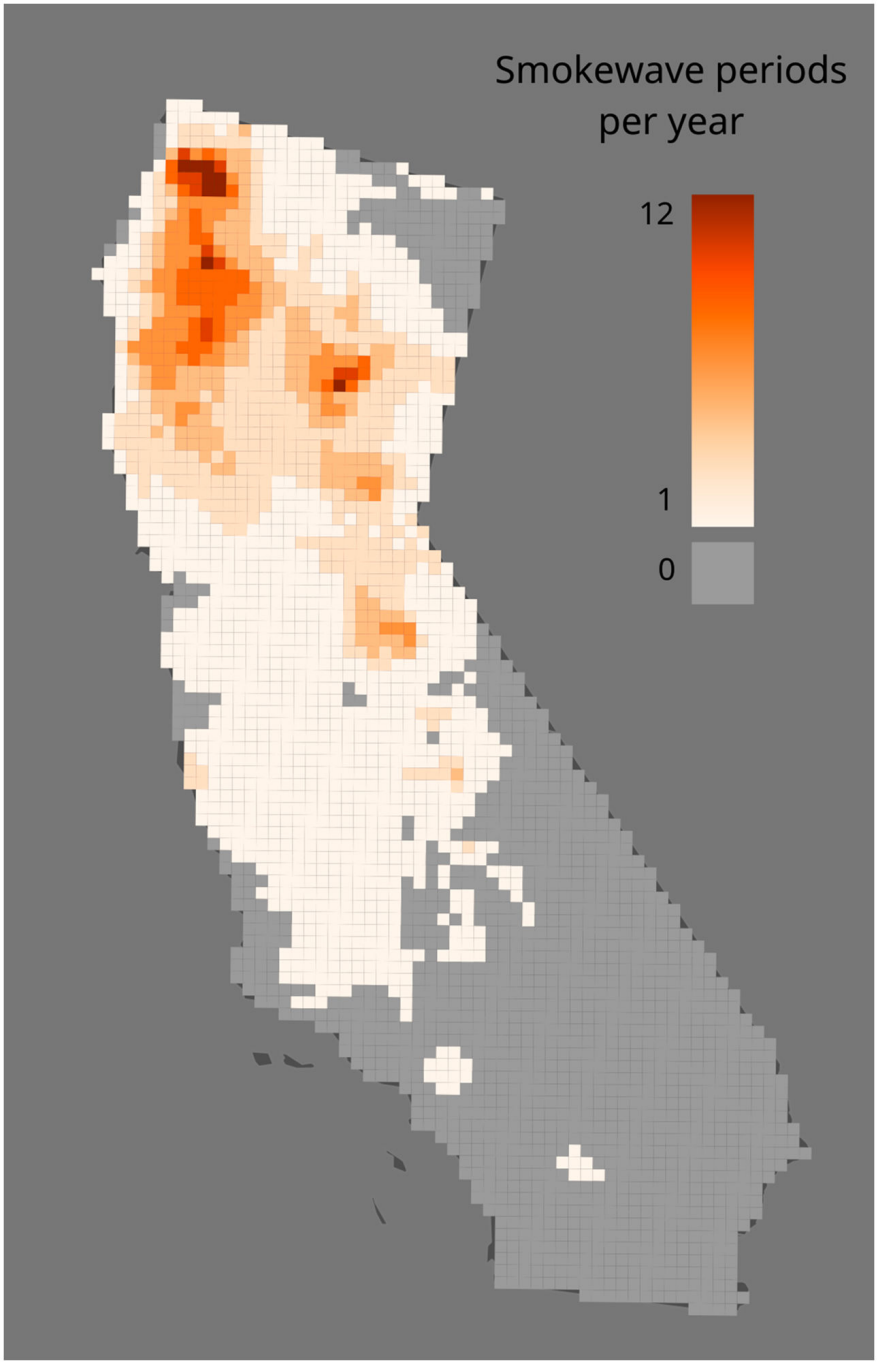
Smokewave periods per year 2007–2013 in California based on CMAQ-modeled fire-PM_2.5_ concentrations. Smokewave periods are defined here as periods when daily fire-PM_2.5_ concentration exceeds the NAAQS 24-h PM_2.5_ standard of 35 μg/m^3^ for more than 2 consecutive days.

Based on the CMAQ-derived fire-PM_2.5_ data, 97.4% of the population of California lived in a county that experienced at least one smokewave from 2007–2013 (Figure 5). A total of 9.2 million individuals (25% of the population of California) lived in a county with at least one smokewave per year on average (i.e., at least seven smokewaves from 2007–2013). Based on a county analysis, a total of 4.5 million individuals (12.2% of the population of California) lived in counties with an average of at least 2 smokewaves per year (i.e., at least 14 from 2007–2013). Although the spatial patterns of wildfires during this period are more concentrated in the northern portion of the state during this period, fires and smokewaves occur statewide in California.

### 3.4. Model Performance

Model skill in replicating smoke impacts was assessed by comparing daily average speciated PM_2.5_ measurements with model predictions when the model predicted wildfire impacts greater than 0.34 μg/m^3^ of PM_2.5_ carbon (organic and elemental) since these components dominate smoke plume composition. Performance was also assessed when the model predicted none or little impact (less than or equal to 0.34 μg/m^3^) from smoke to help frame underlying biases in the modeling system unrelated to wildfires. Daily average mean observed PM_2.5_ carbon (organic mass and elemental), model predicted PM_2.5_ carbon, and the difference between the daily average predictions and observations are presented by year in Table 4 where the model predicts impacts from wildfire and little or no impact from wildfire. The modeling system tends to underestimate organic carbon when little or no wildfire impact was predicted. Performance for capturing wildfire impacts varied from year to year, with some years over-estimating PM_2.5_ carbon (2008 and 2013 both with a bias ~2 μg/m^3^ and one year (2009 with a bias of −2 μg/m^3^) having a notable underprediction (Table 4). CMAQ has been shown to compare reasonably with measurements in urban and rural areas for annual simulations [43,61] and specific episodes [46,62].

## 4. Discussion

In this study we estimated the magnitude of potential exposures of fire-PM_2.5_ and all-source ambient PM_2.5_, and the frequency of smokewave days for fire-PM_2.5_ during a recent period in California. We also compared populations with factors known to modify the risk of adverse PM-related health effects (e.g., age, socioeconomic status, pre-existing conditions like asthma) and estimated population size at risk with respect to the magnitude and frequency of smoke concentrations.

We computed the county level fire-PM_2.5_ by year (2007–2013) and from that full distribution calculated quartiles to describe the potential population exposure. Compared to national county-level multi-year average concentrations (based on cut points ratioed from national standards for all-source PM_2.5_ concentrations), California shows a different pattern than the national profile. Upper end distributions are higher for California (20.3 μg/m^3^ fire-PM_2.5_ compared to 4.58 μg/m^3^ fire-PM_2.5_ in the national study) [35].

### 4.1. The Magnitude of Wildfire Smoke Exposure in California

Wildfires are typically short-lived events that elevate PM_2.5_ to several orders of magnitude over ambient levels. Because of the distribution of many days of near-zero fire-PM_2.5_ and much larger peak concentrations during a fire event, an annual average may mask relevant exposures and other exposure metrics may be relevant to health studies. Our long-term research question seeks to understand to what extent peak regional exposures to wildland fire smoke is associated with increased risk of health effects. Wildland fire sources can contribute to peak values; over half of the summer time ambient measures of 24-h PM_2.5_ from all sources measuring greater than 35 μg/m^3^ in the contiguous U.S. states occur when a smoke plume is present [63]. Further, in the U.S. in situ monitors may miss peak fire-PM_2.5_ exposures because regulatory monitoring of PM is often conducted on a 1-in-3 or 1-in-6-day schedule and the monitoring network is sparse in fire-prone areas. California began using continuous monitors in 2006 and deploys continuous monitors to any population center of concern during a wildfire incident [64].

Atmospheric transport modeling offers the advantage of full geospatial and temporal coverage as well as the ability to isolate pollution originating from fires, but it can be biased due to limitations with key inputs such as emissions and meteorology. The CMAQ model was used to estimate smokewave days and to provide a complete spatial coverage for multiple years. CMAQ, similar to other smoke models, uses locations of known fires to inform emissions inventories – in our case SmartFire [47,48]– to impose the characteristics and amount of fire emissions in place and time. In the modeling, along with emissions from other sources, the atmospheric transport of fire emissions was simulated based on modeled winds and other meteorological inputs. Simulated atmospheric chemical interactions and reactions are also modeled. While complex, these models have had extensive validation for many sources [14,43,65], including some strengths and limitations with regard to wildland fire sources [14,45], as discussed below.

The atmospheric modeling methods used in this study estimate that between 2007 and 2013 population exposure to smoke in the California was extensive with 56.2% of the population living in counties with the highest three quartiles of annual mean fire-PM_2.5_. Similarly, over half of the children (aged 18 and younger) (57.2%), over half of the elderly (aged 65 and older) (56.5%), and over half of those under twice the poverty level resided in California counties with the highest three quartiles of fire-PM_2.5_. Over half of the births, three quarters of the emergency department visits for asthma, and three quarters of the hospital visits for heart attacks occurred in California counties with the highest three quartiles of fire-PM_2.5_, although this analysis does not establish that the exposures preceded these outcomes. Future comparisons of geocoded cases could be matched with modeled concentrations or smokewave metrics to explore associations.

Based on our modeling, 97.4% of California residents lived in a county with at least one smokewave during the 2007–2013 study period. A quarter of the population (24.7%) lived in a county with on average at least one smokewave per year during this period. Of those 12% resided in a county with at least two smokewaves per year during this period. Although the spatial patterns of wildfires during this period are more concentrated in the northern portion of the state during this period, fires and smokewaves occur statewide in California, so with additional years of data, it is possible that many more counties will show high smokewave exposure.

### 4.2. Spatiotemporal Smoke Exposure Approaches

Wildland fire smoke can affect air quality locally and regionally, but it can be difficult to quantify for purposes of studying health impacts [66]. Three main methods have been used in health studies to characterize exposure to wildfire emissions: 1) atmospheric chemical transport modeling, 2) air quality monitoring, and 3) satellite measures of pollutant concentration or density in the atmosphere, data often combined with in-situ monitoring or other models. A combination of these approaches through data fusion, assimilation, or machine learning have also been explored [66]. While several techniques have been assessed, a consensus on best practice has not been established.

Exposure estimation techniques of fire-PM presented in the literature have strengths and limitations. Federal reference or equivalent method air quality monitoring data offer advantages of high-quality data that have been widely used in epidemiologic studies. Typically, measured air pollutant concentrations are mapped from ambient air quality monitors either through spatial interpolation to a grid or simply assigning concentrations from the nearest station(s) to populations. With the exception of the IMPROVE network, most air quality monitoring stations are located in urban and other densely populated areas creating a lack of information for more remote regions where wildland fire-derived pollution can be high. For fire-specific pollution, these gaps in spatiotemporal coverage may result in air quality monitors missing peak fire smoke concentrations that we hypothesize can be an important feature of exposure. For monitored data, source attribution to fires requires extra steps. In contrast to general air pollution, the extent to which stationary monitors represent population exposures to fire-PM has additional uncertainties due to averting behaviors during a fire event, for example. The rapid deployment of air quality monitors during a fire incident introduces logistical challenges such as high cost, siting, remoteness and extent of fire locations, power source for operation, temperature conditions, and uncertainties related to fire movement. Despite these general challenges, California has been in the forefront of rapid deployment of monitors. Alternatively, low-cost and wearable monitors may provide supplemental information, but they require a large number of study participants, lack quality assurance, and lack of validation for health research [13]. Low-cost monitors can provide real-time supplemental field data which may be combined with other techniques.

Atmospheric chemical transport models have been used for decades to understand exposures to air pollutants [15], but the use of these models for all sources and at a large geographic scale for wildland and prescribed fire pollution is relatively new [7,60,67]. Nevertheless, atmospheric modeling has been used in epidemiologic studies to represent exposure [4,67–72], and research has shown atmospheric chemical transport modeling as an effective tool for exploring the impacts and ramifications of wildfire smoke on air quality [4,7,65]. Previous health studies that use atmospheric models have been conducted at coarser geographic scales (e.g., GEOS-Chem) and have considered only fire-derived pollutants rather than fire in conjunction with PM from other sources (e.g., traffic, utilities) when assessing associations with health, which may overestimate the fire-specific exposures [60,73]. Other studies have used atmospheric modeling in conjunction with adjustments from air quality monitoring data, satellite remote sensing data or additional post-processing statistical techniques [68,71,74–79].

All source PM concentrations for broad geographical areas can be inferred from satellite remote sensing [80,81], offering advantages over modeled approaches. The NASA-CALIPSO is an advanced satellite remote sensing system that uses LiDAR sensing to retrieve aerosol optical depth (AOD), extinction profile, and aerosol type at various altitudes; however, it does not provide reliable surface level pollutant concentrations. This and other satellite sensing systems used for characterizing surface air quality are reviewed by Martin [80]. Some researchers have augmented these datasets with ground-level air quality monitoring to address these limitations [82]. Satellite remote sensing of the atmosphere can provide a unique opportunity to understand global and regional scale presence of elevated pollution events, such as wildfires but are limited with regard to local-scale applications. Furthermore, these passive remote sensors cannot directly measure surface PM and instead record AOD, which is a measure of total column aerosol loading, a metric of aerosols from the surface to the top of the atmosphere. Knowledge of atmospheric conditions and assumptions regarding atmospheric stratification conditions provide a way to retrieve the specific metrics of interest, but with uncertainty. Additionally, clouds and other weather patterns can interfere with the measurements resulting in the retrieval algorithms that rely on broad assumptions in order to relate the satellite measurement to surface-level pollution. Optical satellite imaging (e.g., the Multi-angle Imaging SpectroRadiometer–MISR; https://www-misr.jpl.nasa.gov/) has been used to complement atmospheric sensing to derive information on smoke plume structure, including assessing plume height and density and to validate plume rise and trajectory for smoke modeling. Smoke plume images provide an avenue to improve smoke models and for validating smoke dispersion information, but they do not provide surface level pollutant concentrations by themselves [82,83]. The real value of satellite sensing for air quality lies in its use for supporting atmospheric chemical transport models. The state-of-the-art now and into the future is the use of in-situ and satellite-based measurements of PM to adjust and calibrate well-vetted air quality models, such as CMAQ.

### 4.3. Strengths and Limitations

Our study, using the U.S. EPA’s CMAQ platform, provides a comprehensive and consistent way of assessing exposure to fire-PM_2.5_ over broad space and time, for many years for the entire State of California. Using CMAQ allows us to include non-wildfire PM sources, provides full geographic and fine-scale spatiotemporal coverage (e.g., hourly output), includes robust atmospheric chemistry, and isolates fire-specific pollution from primary and atmospherically transformed emissions [42,43,45]. CMAQ has been compared against the routine surface PM monitoring network and field-deployed in situ monitoring data [14,43,65]. At a regional scale, CMAQ-modeled wildfire PM_2.5_ has been shown to compare well to both aircraft plume transects and remotely sensed aerosol optical depth [14], though in close proximity to wildland fires a study of two large wildfires showed a tendency toward overestimation of PM_2.5_ in comparison to a surface monitor network [45]. Model results are limited by the quality of the emission and meteorological inputs. Our model performance results indicate the modeling system tends to overestimate the magnitude of wildfire impacts on PM_2.5_ at routine surface monitor locations. This was most evident for years with large wildfire impacts (e.g., 2007 and 2008) although the model underestimated impacts in 2009 and showed minimal for other years (2011 and 2012) which also had notable wildfire on the landscape (Table 4). Due to lack of robust techniques to quantitatively differentiate measured PM_2.5_ related to wildfire smoke compared from other sources at routine monitor locations, we have not fully evaluated the model for situations when smoke concentrations are present but the model predicts zero fire impact.

Further advantages of our approach are that this modeling platform provides avenues to understand exposures based on the type of fire, the location of fire, and other aspects of pollution not available with air quality monitoring or satellite data alone. Using a model opens opportunities for exploring associations on the basis of place-based and population-based variables. It provides an avenue for assessing future fire scenarios by providing the data for predictive modeling, scenario-based planning, assignment of geocoded health cases to the gridded modeled air quality for epidemiologic assessment, and the vulnerability mapping of populations [67].

Our study has several limitations. A major limitation of estimating health-relevant received dose with atmospheric modeling is that the location and behavior patterns that affect exposure of the population are generally unknown. During a fire event, people may modify their behavior in ways that change the relationship of modeling estimates to air pollution dose in more routine situations – people may evacuate, close windows or spend less time outdoors. Regarding the counts of asthma emergency department visits, births and cardiac hospitalizations, we did not determine if the fire-specific concentrations preceded the event or if any associations existed. This analysis represents a first step in characterizing potential population exposures for further research.

Another limitation is that our CMAQ modeling outputs were not corrected with in situ monitoring data or remote sensing data, which could reduce exposure misclassification [71]. However, given the sparse nature of routine ground measurements and assumptions required for remotely sensed data a simple approach for correcting or modulating the modeled fire predictions could introduce new errors both spatially and temporally. Current studies are underway to improve smoke modeling capability [84] and advance integration of data sources through machine learning methods.

Wildland fire is a permanent and at times beneficial part of the landscape, and exposures are widespread in the U.S. Because of concerns about health risk from air pollution exposures, population exposure to fire-PM should be minimized as a precaution, especially for vulnerable populations. Atmospheric modeling can play a role in the further characterization of the relationships between fire-PM and health risks.

## 5. Conclusions

Widespread and increasing population exposure to wildland fire smoke leads to an urgent need for new techniques to characterize fire-derived pollution for epidemiologic studies. Atmospheric chemical transport modeling is an approach that allows extensive exploration of exposure to fire emissions in space and time. Using CMAQ modeling with and without wildland fire emissions, we found widespread areas in California with fire-related PM_2.5_ concentrations and smokewave days. The 24-h average concentration of PM_2.5_ from all sources in 12-km gridded output from all sources in California (2007–2013) was 4.91 μg/m^3^ (standard deviation 4.04 μg/m^3^). The average concentration of fire-PM_2.5_ in California by year was 1.22 μg/m^3^ (standard deviation 3.78 μg/m^3^). This represents about a quarter of the total ambient PM_2.5_ concentrations. The fire-PM_2.5_ daily mean ranged from 0.31 μg/m^3^ (2010) to 4.40 μg/m^3^ in a high fire year (2008). Although this study focused on years in which the fires were largely in the northern portion of the state, fires and smokewaves occur throughout the state as a norm. Based on the model-derived fire-PM_2.5_ data, 97.4% of the population of California lived in a county that experienced at least one smokewave from 2007–2013, yet the impact on health of smoke is only beginning to be understood. The strengths of our modeling study include the state-of-the-art chemical transport model and full spatial and temporal coverage of fire-PM_2.5_ that cannot be obtained with current in situ air quality monitoring, nor satellite sensing alone. Modeling can isolate and attribute wildland fire sources of PM (including secondarily formed PM) in order to aid causal inference in future health studies. Limitations include the need for a further validation of measurements, including satellite-based PM retrievals. Atmospheric modeling can provide data at a temporal and spatial scale needed to assess exposures for epidemiologic studies, which could be utilized in future work to understand more fully how multi-temporal and broad spatial-scale epidemiological impacts relate to wildland fire smoke exposure.

## Figures and Tables

**Table 1.** Mean daily PM_2.5_ by year for CMAQ 12-km grid cells within California (2007 – 2013).

Year	PM_2.5_ Mean Daily Concentration (Standard Deviation) (μg/m^3^)		Percent Attributable to Fire
	All Sources	Fire Only	
2007	4.62 (2.27)	0.87 (1.55)	18.9%
2008	8.90 (8.76)	4.40 (8.89)	49.4%
2009	4.77(1.50)	0.61 (0.91)	12.7%
2010	4.60 (1.51)	0.31 (0.47)	6.8%
2011	3.90 (1.43)	0.50 (0.70)	12.8%
2012	3.84 (1.51)	0.71 (1.16)	18.4%
2013	3.74 (1.94)	1.16 (1.89)	30.9%
Average	4.91 (4.04)	1.22 (3.78)	24.9%

**Table 2.** Mean daily CMAQ-derived PM_2.5_ by county in California (2007–2013).

County	PM_2.5_ Mean (std) (μg/m^3^)		Percent Attributable to Fire
	All Sources	Fire Only	
Alameda	8.50 (5.90)	0.84 (3.76)	9.9%
Alpine	3.13 (7.63)	1.57(7.55)	50.1%
Amador	6.21 (7.15)	1.87(6.78)	30.1%
Butte	6.61 (13.34)	2.63 (13.14)	39.8%
Calaveras	5.46 (7.06)	1.88 (6.79)	34.4%
Colusa	5.17(8.99)	1.97(8.72)	38.1%
Contra Costa	11.05 (8.29)	0.98 (4.13)	8.9%
Del Norte	4.42 (12.02)	2.74(11.88)	62.0%
El Dorado	5.37 (7.90)	2.23 (7.70)	41.5%
Fresno	5.23 (4.42)	1.10 (3.73)	21.1%
Glenn	5.25 (9.46)	2.04 (9.21)	38.7%
Humboldt	4.63 (11.22)	2.61 (11.09)	56.4%
Imperial	3.46 (1.61)	0.26 (0.64)	7.6%
Inyo	2.28 (2.19)	0.49 (1.43)	21.6%
Kern	4.81 (3.29)	0.76 (2.41)	15.7%
Kings	7.52 (6.63)	0.93 (3.48)	12.3%
Lake	4.37 (10.94)	2.12 (10.83)	48.4%
Lassen	3.30 (6.88)	1.61 (6.78)	48.8%
Los Angeles	8.41 (4.09)	0.57(1.68)	6.8%
Madera	5.45 (4.84)	1.31 (4.39)	24.0%
Marin	4.97 (5.55)	0.84 (3.80)	16.9%
Mariposa	4.47 (8.33)	2.08 (8.24)	46.7%
Mendocino	4.31 (11.45)	2.26 (11.35)	52.5%
Merced	7.40 (6.21)	1.10 (4.36)	14.8%
Modoc	2.74 (4.36)	1.25 (4.20)	45.7%
Mono	2.32 (3.52)	0.82 (3.32)	35.5%
Monterey	3.99 (4.06)	0.81 (3.49)	20.3%
Napa	5.39 (7.85)	1.53 (7.43)	28.4%
Nevada	5.64 (10.48)	2.25 (10.30)	39.9%
Orange	12.09 (6.10)	0.51 (1.57)	4.2%
Placer	6.99 (10.87)	2.41 (10.67)	34.5%
Plumas	4.50 (11.04)	2.43 (10.93)	54.1%
Riverside	4.28 (2.18)	0.34 (0.95)	8.0%
Sacramento	11.83 (9.68)	1.53 (6.50)	13.0%
San Benito	4.12 (3.92)	0.74 (3.12)	17.9%
San Bernardino	3.42 (2.12)	0.36 (0.96)	10.7%
San Diego	5.80 (2.84)	0.40 (1.22)	7.0%
San Francisco	No data	No data	-
San Joaquin	9.72 (7.78)	1.16 (5.07)	12.0%
San Luis Obispo	4.42 (3.47)	0.63 (2.18)	14.4%
San Mateo	6.23 (6.22)	0.70 (3.17)	11.3%
Santa Barbara	3.83 (2.87)	0.64 (2.25)	16.8%
Santa Clara	7.28 (5.37)	0.86 (3.84)	11.8%
Santa Cruz	6.43 (5.38)	0.86 (3.62)	13.3%
Shasta	4.52 (9.12)	2.24 (9.00)	49.4%
Sierra	3.84 (8.48)	1.83 (8.36)	47.6%
Siskiyou	4.24 (9.93)	2.63 (9.87)	62.1%
Solano	8.26 (7.41)	1.25 (5.62)	15.1%
Sonoma	5.37 (8.68)	1.53 (8.32)	28.5%
Stanislaus	7.52 (6.57)	1.17(5.11)	15.6%
Sutter	9.03 (9.51)	1.79 (7.68)	19.9%
Tehama	5.17(12.97)	2.68 (12.88)	51.8%
Trinity	5.10 (19.25)	3.57 (19.20)	70.1%
Tulare	5.11 (3.63)	1.07(3.06)	21.0%
Tuolumne	4.46 (9.68)	2.26 (9.65)	50.7%
Ventura	4.74 (2.95)	0.56 (1.77)	11.8%
Yolo	7.30 (8.68)	1.69 (7.76)	23.1%
Yuba	7.77 (9.38)	2.03 (8.70)	26.2%

**Table 3.** Population size at risk summarized by county and annual average fire-PM_2.5_ in California, 2007–2013.

County Annual Mean Fire-PM_2.5_ (μg/m^3^)	Asthma Emergency Department Visits^a^	Births^b^	Hospitalizations for Heart Attack^a^	Poverty: Under Twice Poverty Line (Poor or Struggling)^c^	Population Under 18^c^	Population 65 and Over^c^	Total Population^c^
Total	2908	591,359	1574	13.67	10.60	4.67	36.78
(0.00, 0.34]	677	241,761	350	5.73	4.44	1.92	17.45
(0.34, 0.56]	489	84,170	235	1.85	1.52	0.65	5.87
(0.56, 0.86]	626	141,995	336	3.37	2.52	1.08	9.77
(0.86,20.3]	1079	114,496	634	2.49	2.02	0.91	7.87
Missing	38	8936	20	0.22	0.11	0.11	0.80

a Asthma emergency department visits and hospitalizations for heart attack is from CDC Environmental Public Health Tracking Network (https://ephtracking.cdc.gov/DataExplorer/#/).

b Births: County-level data is recorded only for counties with populations of 100,000 persons or more. Counties with fewer than 100,000 persons are combined together under the label “Unidentified Counties.” In order to allocate births to those counties, the total number of births of those “Unidentified Counties” were proportionally allocated according to the total population of each county. (Source: https://wonder.cdc.gov/natality.html). Counties with number of births allocated: Alpine County, Yuba County, Amador County, Calaveras County, Colusa County, Del Norte County, Glenn County, Inyo County, Lake County, Lassen County, Mariposa County, Mendocino County, Modoc County, Mono County, Nevada County, Plumas County, San Benito County, Sierra County, Siskiyou County, Sutter County, Tehama County, Trinity County, and Tuolumne County.

c Population size is given in millions, averaged (2007–2013); source of population data is U.S. Census. The poverty level varies by year, size of persons in a family household, and other factors. For example, for 2019, for a family of four in California, the poverty level is $25,750 (2019 dollars), so twice the poverty level is $51,500 per year.

**Table 4.** Annual mean observed, predicted, and difference between observed and predicted PM_2.5_ carbon (organic and elemental components) are shown for each year simulated. Metrics were estimated where modeled wildland fire impacts exceed 0.34 μg/m^3^ (wildland impacted) and otherwise (little or no wildfire).

Type of Wildland Fire Impact	Year	N (Grid Cells)	Mean Observed (μg/m^3^)	Mean Predicted (μg/m^3^)	Difference: Predicted-Observed (μg/m^3^)
Wildfire impacted organic and elemental carbon components of PM_2.5_	2007	463	4.1	4.5	0.5
	2008	721	5.2	7.1	2.0
	2009	422	5.6	3.5	−2.1
	2010	220	3.8	3.0	−0.8
	2011	428	3.5	3.2	−0.3
	2012	418	3.9	3.7	−0.1
	2013	589	3.7	5.3	1.6
Little or no wildfire organic and elemental carbon components of PM_2.5_	2007	918	1.6	1.1	−0.5
	2008	599	1.5	1.5	−0.1
	2009	966	1.4	1.2	−0.2
	2010	1158	1.4	1.2	−0.2
	2011	947	1.3	1.0	−0.4
	2012	1008	1.3	1.0	−0.4
	2013	776	1.2	0.7	−0.5
